# Increased neurotoxic gliosis and blood-brain barrier dysregulation in canine cognitive dysfunction syndrome (CCD)

**DOI:** 10.3389/fragi.2026.1829442

**Published:** 2026-06-02

**Authors:** Sean W. Boland, Greta N. Coyner, Sydney J. Risen, Abdullatif Alsulami, Stephanie McGrath, Julie A. Moreno

**Affiliations:** 1 Department of Environmental and Radiological Health Sciences, College of Veterinary Medicine and Biomedical Sciences, Colorado State University, Fort Collins, CO, United States; 2 Brain Research Center, Colorado State University, Fort Collins, CO, United States; 3 Department of Clinical Sciences, College of Veterinary Medicine and Biomedical Sciences, Colorado State University, Fort Collins, CO, United States; 4 Center for Healthy Aging, Colorado State University, Fort Collins, CO, United States

**Keywords:** astrocytes, blood-brain barrier (BBB), canine cognitive dysfunction, microglia, neuroinflamamation

## Abstract

**Background:**

As Alzheimer’s Disease (AD) and related dementias (ADRD) prevalence is projected to double by 2050, the urgency for relevant models to study its pathology intensifies. Aging is the primary risk factor for AD development and influenced by a myriad of factors including neurotoxic glial activation and BBB degradation; however, historically AD models consist of transgenic mouse *in-vivo* and *in-vitro* methods. While this offers many strengths, it is limited in their ability to mimic aging induced AD pathology. Currently, canine cognitive dysfunction (CCD) syndrome is being investigated as it is a natural and spontaneous disease with similar pathologies to AD and other dementias. Our lab and many others have worked extensively to characterize CCD pathology. However, the role of microglial and astrocytic activation and the interplay between the blood-brain barrier (BBB) have not been investigated. This study aims to fill this gap in knowledge in CCD and in turn its relation to human AD. We hypothesize that CCD-afflicted canines will exhibit increased neurotoxic glial activation and BBB degradation.

**Methods/Results:**

We utilized immunohistochemistry (IHC), morphological analyses, and immunofluorescence to investigate CCD pathology comparing CCD negative and CCD positive dogs. In this study we see glial morphology consistent with those seen in neurotoxic glia in neurodegenerative disease, increased S100β/C3 astrocyte activation, decreased claudin-5 expression, and region-dependent perivascular AQP4 expression modulation in dogs with CCD, compared to those without CCD.

**Conclusion:**

Our results further characterize glial and BBB roles in CCD pathogenesis and reinforces the strengths of modeling AD/ADRD in aging dogs.

## Introduction

Alzheimer’s disease (AD) and related dementias (ADRD) are age-associated neurodegenerative disorders, characterized by progressive cognitive impairment, currently impacting over 7 million Americans, with cases projected to double by 2050. Accumulation of amyloid beta (Aβ) plaques and hyperphosphorylated tau tangles form the classical neuropathological hallmarks of AD (Meadowcroft et al., 2009; Wildburger et al., 2018; Iqbal et al., 2009; Arriagada et al., 1992), which the field utilizes as a target for therapeutic intervention. However, these therapeutic approaches have proven inconsistent with results of patient outcomes (Zhang et al., 2023), leading to investigation in other cellular stress pathways that may play a concurrent role in ADRD pathogenesis. Rising evidence suggests one pathway is neuroinflammation, as it is an important player in multiple neurodegenerative diseases and specifically AD.

Neuroinflammation plays a central role in the development of numerous neurological disorders (Kempuraj et al., 2016). Specifically in AD, growing evidence indicates that neuroinflammation occurs simultaneously with increased accumulation of Aβ plaques and neurofibrillary tau tangles (LaRocca et al., 2021; Carret-Rebillat et al., 2015; Povala et al., 2025; Minter et al., 2016). Neuroinflammation is regulated by glia; particularly, microglia and astrocytes, which exhibit distinct phenotypic plasticity in response to specific physiological and pathological stimuli, resulting in functional outcomes that range from neuroprotective to neurotoxic (Kwon and Koh, 2020). Microglial morphology is highly heterogenous and is region and context dependent. However, generally under normal conditions, microglia adopt a small soma and highly ramified cell processes (Vidal-Itriago et al., 2022; Leyh et al., 2021; Colombo et al., 2022; Milner et al., 2022; Shinozaki et al., 2017). In contrast, pathological stressors such as aging or ischemia induce a transition from resting or protective states into a neurotoxic phenotype (Franco-Bocanegra et al., 2021; Lull and Block, 2013; Tsay et al., 2013; Fernández-Arjona et al., 2017). Neurotoxic reactive microglia are characterized by an ameboid morphology, with enlarged cell bodies and reduced branching compared to its neuroprotective counterparts. Similarly, astrocytes also undergo phenotypic remodeling in response to injury and disease, although the relationship between their morphology and functional state is also region and context-dependent. However, neuroprotective astrocytes generally exhibit hypertrophic remodeling, including increased area, branch number, and complexity (Shinozaki et al., 2017; Delgado-García et al., 2024). Conversely, in neurodegenerative models of AD and Parkinson’s Disease (PD), astrocytes show decreased expression of morphology-related genes, reduced area, branch number, and structural complexity, illustrating a shift toward a neurotoxic phenotype (Endo et al., 2022; Ramos-Gonzalez et al., 2021). Following activation, neurotoxic astrocytes upregulate the expression of glial fibrillary protein (GFAP), S100 calcium-binding protein beta (S100β), and complement component 3 (C3)—and subsequent co-localization being a common feature of their activated state (Marshak et al., 1991; Mori et al., 2010; Zhang et al., 2011; Van Eldik et al., 1994; Chaves et al., 2010; Latham et al., 2024; Hines et al., 2023; Simpson et al., 2010; Cristóvaõ and Gomes, 2019; Liddelow et al., 2017). Activated glial cells subsequently produce pro-inflammatory cytokines and chemokines, including tumor necrosis factor-alpha (TNF-α), interleukin-1 beta (IL-1β), and chemokine ligands 2 and 5 (CCL2 and CCL5). Acute glial activation and neuroinflammation are essential to maintain neuronal homeostasis, such as pathogen elimination and efferocytosis (Chen et al., 2025). However, in pathological states such as aging or disease, these cells can become chronically activated causing chronic neuroinflammation, creating an environment that promotes dysregulation of neuronal and neuro-protective functions, leading to increased neurodegeneration.

Chronic neuro-and-peripheral inflammation contributes to direct neuronal toxicity, and—at the same time, dysfunction of the neuroprotective blood-brain-barrier (BBB) (Rigor et al., 2012; Daniels et al., 2014; Tsao et al., 2021; Beard et al., 2014). The BBB is essential in maintaining neurovascular homeostasis, through tightly coordinated interactions among specialized brain microvascular endothelial cells (BMECs), pericytes, and astrocytes. BMECs form the barrier of the BBB, while astrocytic endfeet provide metabolic and structural support, including regulation of water transport via aquaporin-4 (AQP4) channels (Abbott a al., 2006; Zhang et al., 2019; Jeon et al., 2021; Owasil et al., 2020). BMECs express various tight junction proteins. Claudin-5, the principal tight-junction protein of BMECs, is essential for maintaining BBB selectivity by restricting paracellular diffusion of <800 Da (Da) molecules (Greene et al., 2019; Vázquez-Liébanas et al., 2024; McMillin et al., 2015; Nitta et al., 2003). Although other claudins are expressed at the BBB, they appear to play more modulatory rather than an essential role compared to claudin-5 (Castro Dias et al., 2019a; Castro Dias et al., 2019b). Other essential tight junction proteins that regulate paracellular permeability include occludin and—along with claudin-5, are anchored to actin filaments via zonula occludens (Bolten et al., 1998; Yuan et al., 2020; Abbott a al., 2006; Förster et al., 2008; Sugiyam et al., 2023). The basement membrane further reinforces BBB stability, with collagen IV—its most abundant component providing structural integrity and anchorage (Cottarelli et al., 2020). Dysregulation of collagen IV, claudin-5, and AQP4 each are linked to BBB dysregulation and dementia (Kecheliev et al., 2023; Zeppenfeld et al., 2017; Rabkin, 2023; Zenaro et al., 2015; Zhang et al., 2021; Wisniewski et al., 1997).

Despite clear associations of neuroinflammation and BBB dysregulation with ADRD pathology (Zlokovic, 2014; Nelson et al., 2016; Sweeney et al., 2015; Sengillo et al., 2013; Cullen et al., 2005; Hultman et al., 2013; Miners et al., 2018; Zipser et al., 2007; Halliday et al., 2016; Baloyannis and Baloyannis, 2012; Salloway et al., 2002), limitations in current research models have hindered our full understanding of these mechanisms during natural aging. Current research in the field utilizes transgenic mice to model AD pathology, which offers strengths, but also many limitations. Primary limitations include natural aging and environmental factors association with increased ADRD risk being negated. As aging is the greatest known risk factor for developing ADRD, this creates a significant gap in how the field translates from laboratory rodent animal models to human ADRD pathogenesis.

Canines are increasingly being assessed in the field of ADRD, due to their development of a canine analog of human ADRD called canine cognitive dysfunction (CCD) syndrome (McGrath et al., 2025). Unlike laboratory rodent models, aging canines naturally and spontaneously develop CCD, manifesting a comprehensive neuropathological and behavioral phenotype remarkably similar to AD and other dementias, providing a naturally occurring model to investigate disease progression influenced by age and the environment. Additionally, the genetic diversity within canine models resembles genetic diversity in human disease, creating an additional strength. We and others have shown in previous studies that aged canines have a significant increase in pro-inflammatory microglia activation, neurotoxic astrocyte activation, Aβ plaques, and neurofibrillary tangles, compared to young canines (Prpar Mihevc et al., 2020; Thomsen et al., 2021; Alsulami et al., 2025). However, we have yet to investigate senior canines and the complex interplay between glia, neuroinflammation and BBB modulations in dogs with clinically diagnosed CCD (CCD+) compared to those dogs with no CCD diagnosis (CCD-). To study this, we analyzed the neuro-microenvironment from age-matched senior dogs with CCD+ and CCD- and found disruption in the CCD + dogs. In this study, we hypothesize that dogs with CCD, confirmed through owner survey and Aβ plaque accumulation, will have increased phenotypic shift towards neurotoxic glial, neuroinflammation, and BBB dysregulation. These factors along with plaque and neurofibrillary tangle accumulation contributes to neurodegeneration and subsequent CCD development.

## Materials and methods

### CCD clinical diagnosis and behavioral assessment

CCD status was determined by the owner-survey Canine Dementia Scale (CADES) (Bray et al., 2023; Madari et al., 2015; Simon et al., 2025; Salvin et al., 2011) and Aβ pathology confirmed in our previous study (Alsulami et al., 2025). CCD negative (CCD-) dogs were selected based on a CADES score less than 8 and exhibit negative Aβ pathology. CCD positive (CCD+) dogs were selected based on a CADES score greater than or equal to 20 and are positive for Aβ pathology.

### Sample collection

All canine brains were obtained from privately owned dogs with owner consent after euthanasia. The brains were collected at the Veterinary Teaching Hospital at Colorado State University. Following collection, the brains were fixed in 10% neutral buffer formalin for at least 72 h. After fixation, the brains were coronally sectioned and processed using a tissue processor (Leica TP 1020) and embedded in paraffin wax using a tissue embedder (Leica, E.G., 1160). Blocks containing middle frontal gyrus (cortex) and hippocampal regions were selected for sectioning, staining, and analysis. Using a microtome (ThermoFisher HM1030), sections were cut at 5 μm and mounted on charged slides. We excluded canines that had cognitive comorbidities such as brain tumors or epilepsy. Details regarding age and cognitive scores of the canines are listed in Table 1.

**TABLE 1:** Canines used in study with breed, CADES score, age, and Aβ pathology status.

CCD-canine ID	Breed	CADES score	Age at euthanasia (yrs)	Aβ pathology
Ca169	Mixed breed	0	8	No
Ca264	Dachshund	0	13	No
Ca270	Shetland Sheepdog (Sheltie)	0	4	No
Ca514	Siberian Husky	0	13.5	No
Ca548	Chesapeake Bay Retriever	4	10.2	No
Ca289	Poodle	55	15	Yes
Ca533	Mixed breed	27	10.1	Yes
Ca536	German Shepherd	20	12.25	Yes
Ca541	Mixed breed	93	N/A*	Yes
Ca569	Bichon frise	36	13.3	Yes

* Senior aged dog, but exact age at time of death is unknown.

### Immunohistochemistry (IHC)

Coronal cut tissue sections were deparaffinized by heating slides for 20 min at 60 °C followed by incubation in xylenes and graded ethanol (xylene, 1 part xylene to 1 part 100% EtOH, 100% EtOH, 95% EtOH, 70% EtOH, 1.0 M TBS) for 5 min each. Afterwards they underwent antigen retrieval in 1X EDTA buffer (10 mM EDTA disodium salt dihydrate, 0.05% Tween; pH 9.0) for 20min at 95 °C. Endoperoxidases were inactivated by incubating tissue in 0.3% hydrogen peroxide in H_2_O. Tissue was blocked in TrisA (0.2% Triton-X in 0.05 M TBS) containing 2% bovine serum albumin (BSA, Sigma-Aldrich) and 10% horse serum (Corning) for 1 h (h) at room temperature. Primary antibodies were made in Tris A/2% BSA and incubated overnight in a humidity chamber at 4 °C. The following primary antibodies were used: goat anti-ionized calcium-binding adapter molecule 1(Iba1) at 1:400 dilution (Abcam AB5076) and rabbit anti-GFAP at 1:400 dilution (Dako Z0334). Tissue was washed with Tris A/2% BSA and incubated with biotinylated secondary antibody at 1:250 (Vector Laboratories) for 1h, washed, and incubated with ABC complex (Vector Laboratories) for 1h, as per manufacturer’s instructions, washed, and incubated with DAB (Vector Laboratories) until color change was observed (time dependent on antibody used). Tissue was washed with TBS and counterstained with hematoxylin (Epredia) and bluing reagent (Cancer Diagnostics, Inc). Tissue was dehydrated and coverslips (Globe, #1) were mounted with media (Epredia) and dried overnight or longer before imaging with the Olympus BX53 Scanning Microscope. Representative 20x images were taken using the Olympus BX53.

### Skeletonization analysis

Slides containing canine cortical and hippocampal sections were stained with either anti-Iba1 or anti-GFAP antibodies and imaged at 20x of both CCD+ and CCD-dogs. Ten Iba1+ microglia and GFAP + astrocytes were chosen at random per canine sample for further morphological analysis similar to previous published works (Hay et al., 2025). A series of uniform ImageJ plugin protocols were used to convert individual microglia or astrocytes to binary to quantify cell area, soma area, and circularity (Young and Morrison, 2018). Next, binary images were converted to outlines for fractal analysis to quantify microglia complexity. Last, binary images were skeletonized to examine microglia and astrocyte ramification via branch number and sum of branch lengths. Cell area, soma area, and circularity were quantified for individual astrocytes using the Analyze Particles tool in ImageJ. Fractal analysis was completed with the Fractal Box Count Tool on ImageJ in which Fractal Dimension represents cell complexity. Skeletonized images were processed using the Analyze Skeleton plugin. Summarize Skeleton feature was utilized to calculate the total number of branches and sum of branch lengths for each microglia. N = 50 for each group and brain region (10 cells/canine x 5 canines per group). ROUT outlier analysis and Welch’s Unpaired T-Test was completed for each output, with statistical significance denoted p < 0.05.

### Immunofluorescence (IF)

Coronal cut tissue sections were deparaffinized by heating slides for 20 min at 60 °C followed by incubation in xylenes and graded ethanol (xylene, 1 part xylene to 1 part 100% EtOH, 100% EtOH, 95% EtOH, 70% EtOH, 1.0 M TBS) for 5 min each. Heat- and chemical-induced antigen retrieval was performed by incubating tissue in 1X EDTA buffer (10 mM EDTA disodium salt dihydrate, 0.05% Tween; pH 9.0) for 20 min at 99 °C. Tissue was washed with 0.05 M TBS and blocked using 2% donkey and/or goat serum in TrisA (0.2% Triton-X in 0.05 M TBS) for 1 h at room temperature. After being diluted to their optimized concentrations, primary antibodies were incubated on the tissue at 4 °C overnight in a humidified chamber. BBB integrity was analyzed by detecting collagen IV, claudin-5, and aquaporin-4 (AQP4). Collagen IV was identified using an anti-Collagen IV antibody at a 1:100 concentration (Abcam, Cat #: AB6586) and a donkey anti-rabbit Alexa Fluor 647 secondary antibody (Invitrogen, Cat #: A21244). Claudin-5 was identified using an anti-Claudin-5 polyclonal antibody at a 1:50 concentration (Invitrogen, Cat #: 34–1600) and a donkey anti-rabbit Alexa Fluor 647 secondary antibody (Invitrogen, Cat #: A21244). AQP4 was identified using anti-Aquaporin-4 monoclonal antibody at a 1:100 concentration (ABclonal, Cat #: A11210) and a donkey anti-rabbit Alexa Fluor 647 secondary antibody (Invitrogen, Cat #: A21244). For visualizing astrocytes, three antibodies were used: a chicken anti-GFAP antibody at a 1:200 concentration (Aves Labs, Cat #: GFAP), a rat anti-C3 antibody at a 1:100 concentration (Abcam, Cat #: AB11862), and a rabbit anti-S100β antibody at a 1:100 concentration (Abcam, Cat #: AB41548). The astrocyte stain used the following secondary antibodies: goat anti-chicken Alexa Fluor 488 (Invitrogen, Cat #: A11039), donkey anti-rabbit Alexa Fluor 555 (6441-32), and donkey anti-rat Alexa Fluor 647 (Invitrogen, Cat #: A48272). A detailed table outlining the antibodies and concentrations used is included below (Table 2). Four 10-min wash steps (0.05 M TBS) were followed by incubation with the secondary antibodies at 1:500 concentrations for at least 1 h at room temperature in the dark. Tissue was washed three times for 5 minutes each (0.05 M TBS), and stained with Hoechst (Thermo Scientific, Cat #: 62249) diluted 1:2000 in TBS for 3 minutes followed by three additional washes (1.0 M TBS). Slides were cover slipped with Prolong Diamond Anti-fade mounting medium (Fisher Scientific P36961) allowed to harden for 24–48 h at room temperature and then stored at 4 °C in the dark, until microscopy imaging.

**TABLE 2:** Immunohistochemistry (IHC) and Immunofluorescence (IF) antibodies.

Protein target	Primary antibody and concentration	Catalog	Secondary antibody and concentration	Catalog
Iba1 (IHC)	Goat anti-Iba-1 1:400	Abcam AB5076	Donkey anti-goat 1:250	705-065-147
GFAP (IHC)	Rabbit anti-GFAP 1:400	Dako Z0334	Goat anti-rabbit 1:250	BA-1000
GFAP (IF)	Chicken anti-GFAP 1:200	Aves GFAP	Goat anti-chicken (488) 1:500	A11039
S100β (IF)	Rabbit anti- S100β 1:200	Abcam AB41548	Donkey anti- rabbit (555) 1:500	6441–32
C3 (IF)	Rat anti-C3 1:100	Abcam AB11862	Donkey anti-rat (647) 1:500	A48272
AQP4 (IF)	Rabbit anti-AQP4 1:100	Abclonal A11210	Goat anti-rabbit (647) 1:500	A21244
Claudin-5 (IF)	Rabbit anti-claudin-5 1:50	Invitrogen 34–1600	Goat anti-rabbit (647) 1:500	A21244
Collagen IV (IF)	Rabbit anti-collagen IV 1:100	Abclonal AB6586	Goat anti-rabbit (647) 1:500	A21244

### Immunofluorescent imaging and analysis

A protocol adapted from Latham et al., 2024 was used in this process (Latham et al., 2024). Three images for cortical and hippocampal regions were acquired using an ECHO Resolve fluorescence microscope, using a × 20 objective. All slides stained together were also imaged together with identical exposure times per channel. Three images were taken of each animal per region and marker of Collagen IV, Claudin-5, and AQP4 stained by immunofluorescence and were analyzed. For each micrograph, regions of interest (ROIs) were drawn around individual blood vessels of various sizes and orientations within the tissue. Three-to-five vessels per image per brain region were analyzed, and care was taken to exclude artifacts from the analysis. Mean Cy5 intensity of the proteins within (claudin-5 and collagen IV) or surrounding (AQP4) each vessel were quantified using Qupath (version0.6.0). Perivascular region AQP4 expression was assessed with five ROIs drawn surrounding separate vessels and quantified based on the mean Cy5 intensity. Reactive astrocytes were quantified by analyzing C3 co-expression within S100β^+^ astrocytes. This was performed by using manual thresholding on the positive cell detection function of Qupath (6.0) to identify S100β^+^ Hoechst^+^ C3^+^ cells, which were then converted into individual ROIs. Any false negative/positive cells were manually excluded. Afterwards, the percentage of S100β^+^ Hoechst^+^ C3^+^ cells were assessed relative to S100β^+^ Hoechst^+^ cells in each brain region with the following calculation: [(S100β^+^ Hoechst^+^ C3^+^ cells/ S100β^+^ Hoechst^+^ cells) *100]. Furthermore, we assessed C3 mean grey intensity in S100β^+^ Hoechst^+^ C3^+^ cells by randomly selecting 10 S100β^+^ Hoechst^+^ C3^+^ cells and using mean intensity feature in Qupath (6.0). ROUT outlier analysis and Welch’s Unpaired T-Test was completed for each output, with statistical significance denoted p < 0.05.

## Results

### Microglia and astrocytes adopt morphology of neurotoxic phenotypes in CCD + dogs

**FIGURE 1:**
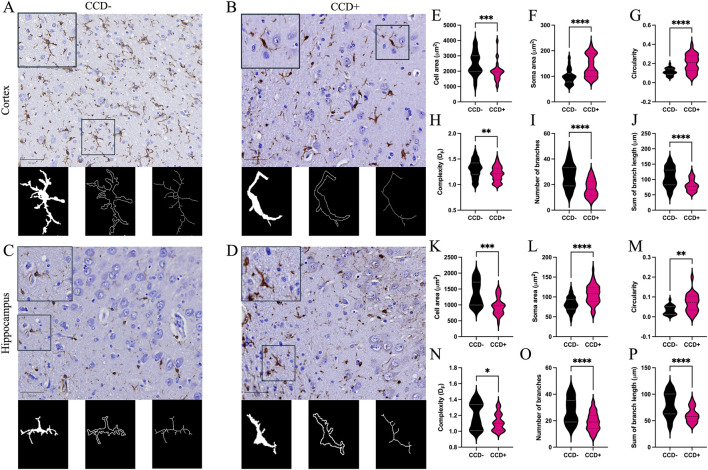
Microglial morphology is significantly altered in the frontal cortex and hippocampus of CCD^+^ canines. Representative Iba1-immunostained images of frontal cortex from CCD^−^ **(A)** and CCD^+^ **(B)** canines and hippocampus from CCD^−^ **(C)** and CCD^+^ **(D)** canines. Insets indicate representative microglia selected for morphological analysis. Ten Iba1^+^ microglia were randomly selected per animal for quantitative analysis (n = 5 animals per group; 50 cells per group). Individual microglia were processed in ImageJ to generate binary masks, outlines, and skeletonized images for morphometric analysis. Binary masks were used to quantify total cell area and soma area. Outline images were used to assess circularity. Skeletonized images were analyzed to determine total branch number and cumulative branch length, while fractal box-count analysis was used to quantify cellular complexity (fractal dimension). Quantification of cortical microglia is shown in panels **(E–J)**, and hippocampal microglia in panels **(K–P)**. Data are presented as mean ± SD (50 cells per group). ROUT outlier detection and unpaired two-tailed t-tests were used for statistical analysis. p < 0.05, p < 0.01, *p < 0.001, **p < 0.0001. Scale bar = 50 μm (main images), 20 μm (magnified images).

**FIGURE 2:**
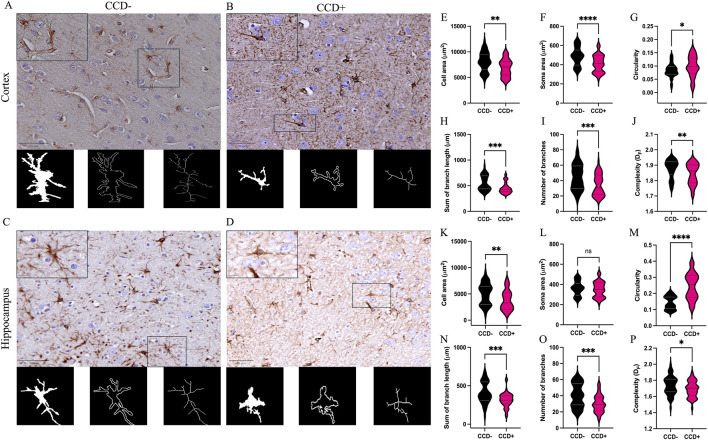
Astrocyte morphology is significantly altered in the frontal cortex and hippocampus of CCD^+^ canines. Representative GFAP-immunostained images of frontal cortex from CCD^−^ **(A)** and CCD^+^ **(B)** canines and hippocampus from CCD^−^ **(C)** and CCD^+^ **(D)** canines. Insets indicate representative astrocytes selected for morphological analysis. Ten GFAP^+^ astrocytes were randomly selected per animal for quantitative analysis (n = 5 animals per group; 50 cells per group). Individual astrocytes were processed in ImageJ to generate binary masks, outlines, and skeletonized images Binary masks were used to quantify total cell area and soma area. Outline images were used to assess circularity. Skeletonized images were analyzed to determine total branch number and cumulative branch length, while fractal box-count analysis was used to quantify cellular complexity (fractal dimension). Quantification of cortical astrocytes is shown in panels **(E–J)**, and hippocampal astrocytes in panels **(K–P)**. Data are presented as mean ± SD (50 cells per group). ROUT outlier detection and unpaired two-tailed t-tests were used for statistical analysis. p < 0.05, p < 0.01, *p < 0.001, **p < 0.0001. Scale bars = 50 μm (main images), 20 μm (magnified images).

To assess neurotoxic microgliosis we performed immunohistochemistry (IHC) on cortical and hippocampal brain tissue regions for CCD- and CCD + dogs (Figure 1). Representative images of IHC staining for microglial marker Iba1 in cortical (Figures 1A,B) and hippocampal (Figures 1C,D) regions are shown, along with binary, outline, and skeleton visualization. Additionally, representative images of astrocytic marker GFAP (Figure 2) in cortical (Figures 2A,B) and hippocampal (Figures 2C,D) are shown, along with cell binary, outline, and skeleton visualization. Staining was followed by detailed morphological analysis using ImageJ skeletonization and fractal analysis. Microglia and astrocytes both adopt morphological characteristics of neurotoxic phenotypes in the cortex and hippocampus of CCD + dogs. Cortical microglia in CCD + dogs exhibited decreased cell area (Figure 1E; −502.4 ± 135.0 SEM p = 0.0003), increased soma area (Figure 1F; 41.62 ± 7.276 SEM p < 0.0001) and circularity (Figure 1G; 0.08848 ± 0.01381 SEM p < 0.0001). These changes were accompanied by reduced structural complexity, including decreased fractal complexity (Figure 1H; −0.08884 ± 0.03208 SEM p = 0.0068), branch number (Figure 1I; −9.640 ± 1.606 p < 0.0001), and sum of branch length (Figure 1J; −25.98 ± 5.250 SEM p < 0.0001) compared to CDD-dogs. A similar pattern was observed in the hippocampus. CCD + hippocampal microglia exhibited decreased cell area (Figure 1K; 24.42 ± 4.204 SEM p = 0.0002), increased soma area (Figure 1L; −386.0 ± 94.57 SEM p < 0.0001) and circularity (Figure 1M; 0.03651 ± 0.01021 SEM p = 0.0011), as well as decreased fractal complexity (Figure 1N; −0.08128 ± 0.03830 SEM p = 0.0397), branch number (Figure 1O; 8.340 ± 1.658 SEM p < 0.0001), and sum of branch length (Figure 1P; −18.70 ± 3.800 SEM p < 0.0001) compared to CDD-dogs.

As for astrocytic morphology, cortical astrocytes in CCD + dogs exhibited decreased cell area (Figure 2E; −1197 ± 356.8 SEM p = 0.0011), soma area (Figure 2F; −76.23 ± 18.41 SEM p < 0.0001), complexity (Figure 2H; −0.03412 ± 0.01264 SEM p = 0.0082), branch number (Figure 2I; −10.42 ± 3.005 SEM p = 0.0008), and the sum of the branch length (Figure 2J; −97.41 ± 25.15 SEM p = 0.0002) compared to CDD-dogs. Additionally, soma circularity increased (Figure 2G; 0.01471 ± 0.006641 SEM p = 0.0295) in CCD + compared to CDD-dogs. Furthermore, hippocampal astrocytic architecture showed a similar trend. CCD + dogs exhibited decreased cell area (Figure 2K; −1236 ± 386.8 SEM p = 0.0019), branch number (Figure 2O; −9.900 ± 2.517 SEM p = 0.0002), sum of branch length (Figure 2P; −91.08 ± 23.11 SEM p = 0.0002), and complexity (Figure 2N; −0.04448 ± 0.02127 SEM p = 0.0391), while also having no significant change in soma area (Figure 2L), but an increase in soma circularity (Figure 2M; 0.1007 ± 0.01265 SEM p < 0.0001).

### Neurotoxic S100β^+^C3^+^ astrocytes and C3 expression are increased in the cortex and hippocampus of CCD^+^ canines

**FIGURE 3:**
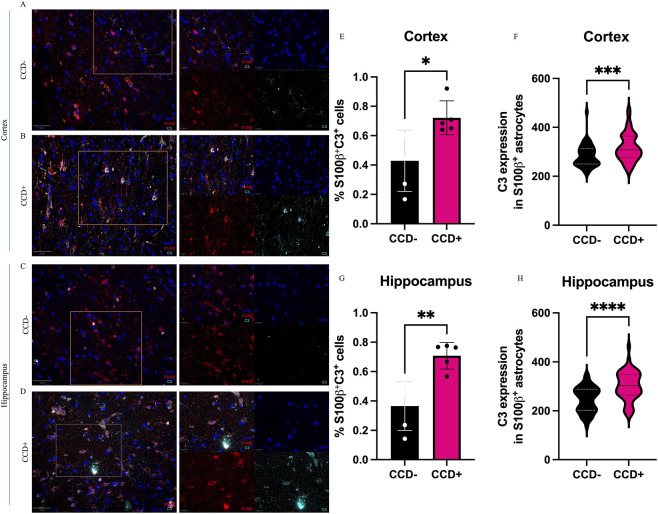
Neurotoxic S100β^+^C3^+^ astrocytes are increased in the cortex and hippocampus of CCD^+^ canines. Representative immunofluorescence images of cortical tissue from CCD^−^ **(A)** and CCD^+^ **(B)** canines and hippocampal tissue from CCD^−^ **(C)** and CCD^+^ **(D)** canines (n = 5 per group). Low-magnification panels show merged Hoechst (blue), S100β (red), and C3 (cyan) labeling. Boxed regions are displayed at higher magnification to highlight representative S100β^+^ astrocytes and C3 co-localization. The percentage of S100β^+^ astrocytes co-expressing C3 was quantified in the cortex **(E)** and hippocampus **(G)**. CCD^+^ canines exhibited a significantly higher proportion of S100β^+^C3^+^ astrocytes in both regions compared with CCD^−^ controls. Relative C3 fluorescence intensity within S100β^+^ astrocytes was additionally quantified in the cortex **(F)** and hippocampus **(H)**, with mean grey intensity per cell, demonstrating increased C3 expression in CCD^+^ canines. Data for **(E,G)** are presented as mean ± SD, with each data point representing the average of three images per animal unless otherwise indicated; and data for **(F,H)** are presented with violin plots of 50 cells per group. Statistical significance was determined using Welch’s two-tailed t-test. p < 0.05 (*), p < 0.01 (**), *p < 0.001, **p < 0.0001. Scale bars = 50 μm (main images), 20 μm (magnified images).

To further characterize neurotoxic astrogliosis, we performed S100β/C3 immunofluorescent co-staining (Liddelow et al., 2017), which is a technique we have utilized in previous studies (Latham et al., 2024; Hines et al., 2023; Hay et al., 2025). Staining was performed on cortical and hippocampal brain tissue regions for CCD- and CCD + dogs (Figure 3). Representative images include cortical and hippocampal for CCD- (Figures 3A,C) and CCD+ (Figuress 3B,D) canines with Hoechst, S100β, C3, and merged images. CCD + canines exhibited a significantly higher proportion of S100β^+^ astrocytes co-expressing C3 compared to CCD + controls in both brain regions. Quantitative analysis confirmed a significant increase in the percentage of S100β^+^C3^+^ astrocytes in the cortex (Figure 3E; 0.2923 ± 0.1068 SEM *p* = 0.0327) and a more pronounced increase in the hippocampus (Figure 3G; 0.3425 ± 0.08452 SEM p = 0.0063). Additional analysis of C3 mean intensity revealed increased expression of C3 in the cortex (Figure 3F; 55.86 ± 11.02 SEM p = 0.0010) and hippocampus (Figure 3H; 55.86 ± 11.02 p < 0.0001) in CCD + dogs. These results indicate regionally consistent enrichment of a neurotoxic astrocyte phenotype in CCD + canines.

### Perivascular AQP4 expression is differentially altered in CCD^+^ canines

**FIGURE 4:**
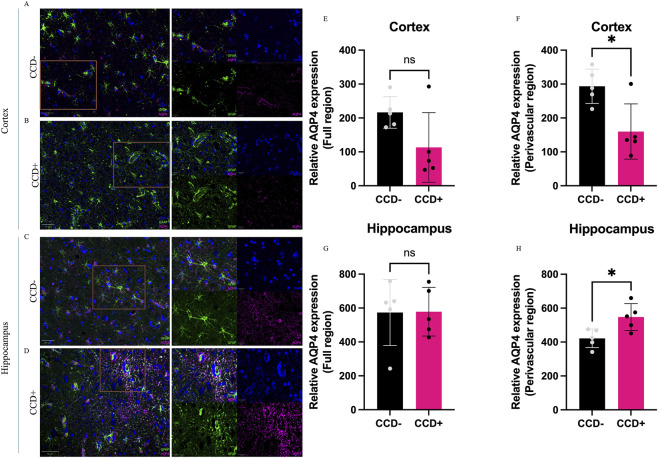
Perivascular AQP4 expression is differentially altered in cortical and hippocampal regions of CCD^+^ canines. Representative immunofluorescence images of cortical tissue from CCD^−^ **(A)** and CCD^+^ **(B)** canines and hippocampal tissue from CCD^−^ **(C)** and CCD^+^ **(D)** canines (n = 5 per group). Low-magnification panels show merged Hoechst (blue), GFAP (green), and AQP4 (magenta) labeling. Boxed regions are displayed at higher magnification to highlight representative microvessels and surrounding astrocytic endfeet. Full-image mean grey intensity per surrounding vessel was quantified to assess total regional AQP4 expression in the cortex **(E)** and hippocampus **(G)**. Relative total AQP4 expression did not differ significantly between CCD^−^ and CCD^+^ canines in either region. For perivascular analysis, five vessels were selected per image and mean grey intensity surrounding each vessel was quantified. CCD^+^ canines exhibited significantly decreased perivascular AQP4 expression in the cortex (**F**; *p* = 0.0250) and significantly increased perivascular AQP4 expression in the hippocampus (**H**; *p* = 0.0192) compared with CCD^−^ controls. Data are presented as mean ± SD, with each data point representing the average of three images per animal. Statistical significance was determined using Welch’s two-tailed *t*-test. *p* < 0.05 (*). Scale bar = 50 μm (main images), 20 μm (magnified images).

To assess whether astrocytic endfoot-associated AQP4 expression was altered in CCD, AQP4 immunoreactivity was quantified in cortical and hippocampal regions of CCD- and CCD + aged canines. Representative images include cortical and hippocampal for CCD- (Figures 4A,C) and CCD+ (Figures 4B,D) canines with Hoechst, GFAP, AQP4, and merged images. Total AQP4 expression did not differ significantly between CCD- and CCD + animals in either the cortex (Figure 4E) or hippocampus (Figure 4G). In contrast, neurovascular-specific analysis revealed significant alterations in perivascular AQP4 expression; CCD + canines exhibited a significant reduction in perivascular AQP4 expression in the cortex (Figure 4F, −133.6 ± 42.92 SEM *p* = 0.0180) alongside a significant increase in perivascular AQP4 expression in the hippocampus (Figure 4H, 125.8 ± 43.01 SEM *p* = 0.0217) compared to CCD-controls. These findings indicate region-specific modulation of astrocytic AQP4 localization in CCD + canines. Due to perivascular disruption, next we investigated BBB integrity.

### Claudin-5 expression downregulated in cortical and hippocampal regions of CCD + dogs neurovascular

**FIGURE 5:**
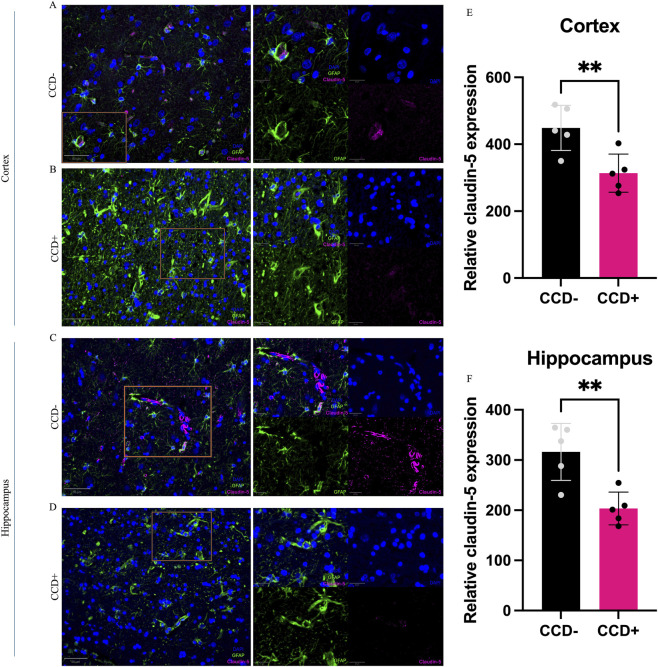
Claudin-5 expression is reduced in cortical and hippocampal microvessels of CCD^+^ canines. Representative immunofluorescence images of cortical tissue from CCD^−^ **(A)** and CCD^+^ **(B)** canines and hippocampal tissue from CCD^−^ **(C)** and CCD^+^ **(D)** canines (n = 5 per group). Low-magnification panels show merged Hoechst (blue), GFAP (green), and claudin-5 (magenta) labeling. Boxed regions are displayed at higher magnification to highlight representative cerebral microvessels and associated claudin-5 localization. For quantification, three vessels were selected per image and mean grey intensity per vessel was measured. CCD^+^ canines exhibited significantly reduced relative claudin-5 expression in both the cortex (**E**; 30.0% reduction, p = 0.0092) and hippocampus (**F**; 35.7% reduction, p = 0.0049) compared with CCD^−^ controls. Data are presented as mean ± SD, with each data point representing the average of three images per animal. Statistical significance was determined using Welch’s two-tailed t-test. p < 0.01 (**). Scale bar = 50 μm (main images), 20 μm (magnified images).

To assess BBB tight-junction integrity, we quantified claudin-5 expression in cortical and hippocampal micro vessels of CCD+ and CCD-canines (Figure 5). Representative images include cortical and hippocampal for CCD- (Figures 5A,C) and CCD+ (Figures 5B,D) with Hoechst, GFAP, claudin-5, and merged images. In CCD-dogs, claudin-5 labeling appeared continuous and closely associated with microvascular structures (Figures 5A,C), whereas CCD + dogs displayed visibly reduced and discontinuous claudin-5 intensity (Figures 5B,D). Quantitative analysis confirmed a significant decrease in CCD + relative claudin-5 expression in both the cortex (Figure 5E; 30.0% reduction p = 0.0092) and hippocampus (Figure 5F; 35.7% reduction p = 0.0049), compared to CCD-dogs. Given these tight-junction deficits, we next assessed whether basement membrane structure was similarly altered.

### Basement membrane collagen IV expression is preserved in CCD + canines

**FIGURE 6:**
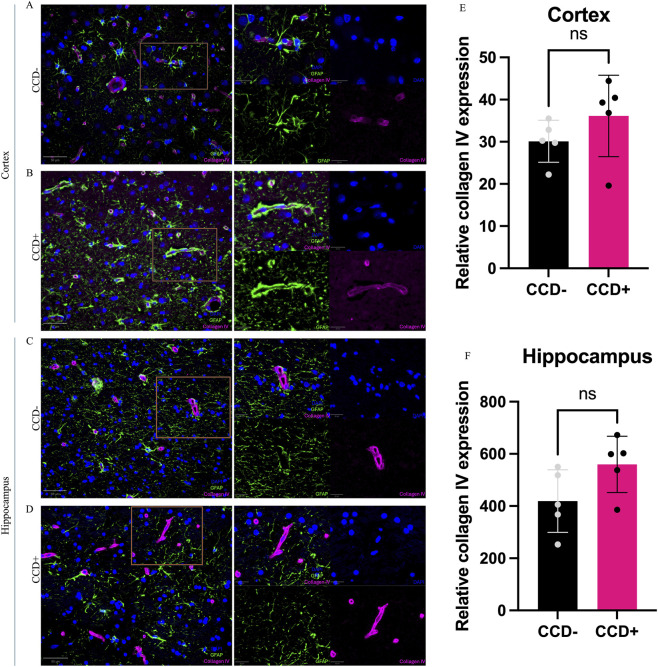
Collagen IV expression is preserved in cortical and hippocampal microvessels of CCD^+^ canines. Representative immunofluorescence images of cortical tissue from CCD^−^ **(A)** and CCD^+^ **(B)** canines and hippocampal tissue from CCD^−^ **(C)** and CCD^+^ **(D)** canines (n = 5 per group). 20x panels show merged Hoechst (blue), GFAP (green), and collagen IV (magenta) labeling. Boxed regions are shown to highlight representative microvessels and surrounding astrocytic processes. For quantification, four vessels were selected per image and mean grey ntensity per vessel was measured. Relative collagen IV expression did not differ significantly between CCD^−^ and CCD^+^ canines in either the cortex (**E**; *p* = 0.2614) or hippocampus (**F**; *p* = 0.0877). Data are presented as mean ± SD, with each data point representing the average of three images per animal. Statistical significance was determined using Welch’s two-tailed *t*-test. Scale bar = 50 μm (main images), 20 μm (magnified images).

To determine whether basement membrane integrity was altered in CCD, collagen IV expression was examined in cortical and hippocampal tissue from CCD- and CCD + dogs using immunofluorescence. Representative images include cortical and hippocampal for CCD- (Figures 6A,C) and CCD+ (Figures 6B,D) with Hoechst, GFAP, collagen IV, and merged images. Quantitative analysis revealed no significant change in relative collagen IV expression in the cortex (Figure 6E) or hippocampus (Figure 6F) of CCD + canines compared to CCD-controls (ns). These findings indicate that, despite alterations in tight-junction protein expression, basement membrane collagen IV levels remain preserved in CCD + canines.

## Discussion

Our group has worked extensively to characterize CCD pathology. In previous studies, we identified increased Aβ plaques, along with hyperphosphorylated tau tangles (). Additionally, we see decreased levels of Aβ_1-42/40_ in the cerebrospinal fluid (CSF) (Alsulami et al., 2025), matching human AD pathology. Furthermore, we identified increased S100β^+^C3^+^ astrocytes in aged canines, compared to young canines (Hines et al., 2023), but we had not investigated between CCD- and CCD + dogs until now. Importantly, no studies to date have investigated glial morphology to characterize an activated state, S100β^+^ C3^+^ astrogliosis, and assess the interplay between astrocytic-BBB dysregulation and CCD pathogenesis.

In this study, we demonstrate that CCD is associated with glial remodeling and BBB alterations that reflect functional dysregulation. CCD + dogs exhibited increased neurotoxic astrocyte activation and microglial morphological reactivity, accompanied by disruption of endothelial tight-junction protein claudin-5 and region-specific alterations in perivascular AQP4, while basement membrane collagen IV expression remained preserved.

Astrocytes and microglia play a central role in maintaining central nervous system homeostasis, yet chronic activation of these cells contributes to sustained neuroinflammation and neuronal vulnerability during aging and neurodegenerative disease (Endo et al., 2022; Ramos-Gonzalez et al., 2021; Marshak et al., 1991; Mori et al., 2010; Zhang et al., 2011; Van Eldik et al., 1994; Chaves et al., 2010; Hines et al., 2023; Simpson et al., 2010; Cristóvaõ and Gomes, 2019; Liddelow et al., 2017; Prpar Mihevc et al., 2020; Thomsen et al., 2021; L’Episcopo et al., 2011). Currently, studies show S100β and C3 are upregulated individually in AD (Mori et al., 2010; Chaves et al., 2010; Cristóvaõ and Gomes, 2019). Our lab has shown increased percentage of astrocytes positive for both S100β and C3 indicating astrocytic activation previously in canines; however, we compared aged vs. young dogs rather than CCD status (Hines et al., 2023). In this study, the increased prevalence of S100β^+^C3^+^ astrocytes, along with increased astrocytic C3 expression and their architectural changes observed in CCD + canines is consistent with a neurotoxic astrocyte phenotype (Figure 3), previously implicated in many neuropathological studies (Latham et al., 2024; Hines et al., 2023; Liddelow et al., 2017; Hay et al., 2025). Concurrent microglial morphological changes further support a shift toward a reactive neuroinflammatory environment and further supporting microglial reactivity occurrence in CCD, as previously reported (Yang et al., 2017)

Astrocytic endfeet play a critical role in Aβ clearance, BBB regulation, and water homeostasis through polarized expression of AQP4. While total AQP4 expression remained unchanged in CCD + canines, perivascular AQP4 exhibited region-specific alterations in the cortex and hippocampus (Figure 4). In the cortex, decreased AQP4 perivascular expression in CCD + dogs is consistent with AQP4 modulation in AD and limits the clearance of Aβ through glymphatic network (Owasil et al., 2020; Zeppenfeld et al., 2017). Whereas in the hippocampus, CCD + dogs displayed increased perivascular AQP4 expression, which is seen in TBI (Jeon et al., 2021). This is a discrepancy based on the majority of current AD literature, which characterizes loss of perivascular AQP4 expression during disease. However, to our knowledge, there is no current literature assessing AQP4 expression in canines and this discrepancy may be attributed to a variety of variables such as the species and/or the stage of the disease. It is possible there is an initial compensatory upregulation of AQP4, followed by depletion; however, this is unclear and requires further investigation. Our findings suggest that CCD is associated with region-specific redistribution of AQP4, potentially reflecting disrupted astrocyte polarity or compensatory remodeling in response to regional vascular or metabolic demands. Such compartment-specific changes may have important implications for neurovascular coupling and fluid homeostasis during aging.

BBB dysfunction is increasingly recognized as a contributor to neurodegenerative disease progression. In the present study, reduced claudin-5 expression in CCD + dogs indicates compromised endothelial tight-junction integrity, which may permit dysregulated paracellular permeability (Figure 5), as seen in rodent BBB models (Greene et al., 2019; Vázquez-Liébanas et al., 2024; McMillin et al., 2015; Nitta et al., 2003; Shibly et al., 2023; Keaney et al., 2015). In contrast, collagen IV expression remained unchanged across regions (Figure 6), suggesting that basement membrane structure is preserved despite endothelial and astrocytic alterations. This dissociation supports emerging evidence that BBB dysfunction during aging may arise from selective molecular vulnerabilities rather than uniform degradation of all barrier components.

Investigation of BBB dysfunction in CCD remains limited. Merbl and their colleague’s employed contrast enhanced with magnetic resonance imaging (MRI) with subtraction enhancement analysis to assess whole-brain BBB permeability and structural atrophy in clinically classified CCD dogs. They reported no significant change in permeability compared to controls (Merbl et al., 2025). Their approach provides valuable macrostructural and permeability insights; however, MRI-based analyses primarily detect overt dye leakage. In contrast, our study utilized postmortem molecular and histological analyses to interrogate specific neurovascular unit components, including claudin-5, collagen IV, and AQP4. Additionally, our cohort incorporated neuropathological assessment of amyloid deposition in addition to CADES assessment, allowing molecular grouping of CCD status. These methodological differences suggest that subtle, region-specific neurovascular alterations may be present in CCD even in the absence of macroscopic permeability changes detected by contrast MRI, and/or a difference in pathology in early vs. late stages of disease.

The use of aged canines as a naturally occurring model of cognitive decline provides a unique opportunity to investigate neurodegenerative mechanisms that incorporate aging, environmental exposure, and genetic diversity. Unlike transgenic rodent models, canines spontaneously develop cognitive dysfunction and neuropathology that closely parallel human Alzheimer’s disease, including amyloid deposition, tau pathology, and neuroinflammatory changes. Our findings reinforce the translational relevance of CCD as a model for studying age-related BBB dysfunction and neuroinflammation in a physiologically relevant context.

While we believe our study offers unique strengths, several limitations should be considered. As mentioned previously, glial morphology is complex, as it differs by region and context. Additionally, GFAP has been shown to only allow for 15% visualization of astrocytic morphology. These key factors highlight limitations in our methods of morphology analysis. However, the technological necessities to perform these updated analytical methods are beyond the scope of this study; and, we believe our results will add vital knowledge to the field, driving further investigation into glial morphology in CCD. Another limitation includes our focus on protein expression and co-localization and not directly assessing BBB permeability, which is an avenue we want to investigate in future studies. Additionally, structural changes in basement membrane organization or tight-junction architecture were not evaluated and may occur despite preserved collagen IV expression. Furthermore, while claudin-5 is the principal barrier, we did not investigate other integral BBB proteins such as zonula occludens, occludin, and PECAM-1.

In conclusion, our data identify selective BBB component disruption, gliosis and astrocytic endfoot remodeling as key features of CCD. Although this is a small subset of aging canines, these significant findings suggest that age-associated cognitive dysfunction in canines is characterized by neurovascular unit modulation driven by glial–endothelial dysfunction. However, the mechanistic intricacies remain unclear and requires further investigation.

## Data Availability

The original contributions presented in the study are included in the article/supplementary material, further inquiries can be directed to the corresponding author.
